# Impact of bone and cartilage segmentation from CT and MRI on both bone forearm osteotomy planning

**DOI:** 10.1007/s11548-023-02929-8

**Published:** 2023-05-23

**Authors:** Ruurd J. A. Kuiper, Joost W. Colaris, Filip Stockmans, Eline M. van Es, Max A. Viergever, Peter R. Seevinck, Harrie Weinans, Ralph J. B. Sakkers

**Affiliations:** 1 Department of Orthopaedics, University Medical Center Utrecht, Heidelberglaan 100, 3584 CX Utrecht, The Netherlands; 2 Image Sciences Institute, University Medical Center Utrecht, Utrecht, The Netherlands; 3 Department of Orthopaedics and Sports Medicine, Erasmus Medical Centre, Rotterdam, The Netherlands; 4 Muscles & Movement, Department of Development and Regeneration, KU Leuven Campus Kulak, Kortrijk, Belgium

**Keywords:** Orthopaedic planning, Osteotomy, Bone, CT, MRI

## Abstract

**Introduction:**

The use of MRI scans for pre-operative surgical planning of forearm osteotomies provides additional information of joint cartilage and soft tissue structures and reduces radiation exposure in comparison with the use of CT scans. In this study, we investigated whether using 3D information obtained from MRI with and without cartilage information leads to a different outcome of pre-operative planning.

**Methods:**

Bilateral CT and MRI scans of the forearms of 10 adolescent and young adult patients with a unilateral bone deformation were acquired in a prospective study. The bones were segmented from CT and MRI, and cartilage only from MRI. The deformed bones were virtually reconstructed, by registering the joint ends to the healthy contralateral side. An optimal osteotomy plane was determined that minimized the distance between the resulting fragments. This process was performed in threefold: using the CT and MRI bone segmentations, and the MRI cartilage segmentations.

**Results:**

Comparison of bone segmentation from MRI and CT scan resulted in a 0.95 ± 0.02 Dice Similarity Coefficient and 0.42 ± 0.07 mm Mean Absolute Surface Distance. All realignment parameters showed excellent reliability across the different segmentations. However, the mean differences in translational realignment between CT and MRI bone segmentations (4.5 ± 2.1 mm) and between MRI bone and MRI bone and cartilage segmentations (2.8 ± 2.1 mm) were shown to be clinically and statistically significant. A significant positive correlation was found between the translational realignment and the relative amount of cartilage.

**Conclusion:**

This study indicates that although bone realignment remained largely similar when using MRI with and without cartilage information compared to using CT, the small differences in segmentation could induce statistically and clinically significant differences in the osteotomy planning. We also showed that endochondral cartilage might be a non-negligible factor when planning osteotomies for young patients.

**Supplementary Information:**

The online version contains supplementary material available at 10.1007/s11548-023-02929-8.

## Introduction 

Radial and ulnar bone malunion after trauma can lead to reduced range of motion, chronic pain, and loss of strength [1]. Studies have shown that these complications can be effectively treated with realignment of the bones by a corrective osteotomy [2, 3]. Corrective osteotomies are conventionally planned and assessed manually on (biplanar) two-dimensional (2D) radiographs [4]. However, recent studies have shown that computer-assisted pre-operative planning based on three-dimensional (3D) computed tomography (CT) scans significantly improved both functional and radiographic outcomes [4–6].

Three-dimensional computer-assisted osteotomy planning is mostly performed using CT scans, as the unique intensity range of bone structures on this modality makes segmentation relatively straightforward and highly accurate (< 1 mm) [7, 8]. However, it is difficult to distinguish between different soft tissue structures on CT, and CT scanning involves harmful radiation. In contrast, osteotomy planning using magnetic resonance imaging (MRI) does not involve harmful radiation and soft tissue structures can be more clearly distinguished. Including soft tissues structures like joint cartilage might improve the outcome of pre-operative planning. However, most MRI protocols do not result in a unique intensity range for bone and can suffer from intensity inhomogeneity, which causes segmentation to be less trivial [9].

To be able to study differences in osteotomy planning based on CT and MRI, a deterministic workflow is essential to avoid the variation caused by human assessment. Various authors have proposed automatic osteotomy planning methods [7, 10, 11]. Based on these methods, we aimed to develop a similar deterministic, pre-operative, and osteotomy planning algorithm that simulates the cutting, repositioning, and reconstruction of the deformed bone. By using an automatic, computer-assisted approach, we attempted to determine the effect of different segmentations from different modalities on the outcome, by finding an answer to the following questions: (1) Does automated osteotomy planning based on bone segmentations derived from CT scans yield significantly different results when performed on bone segmentations derived from MRI scans? (2) Does automated osteotomy planning based on bone segmentations derived from MRI scans yield significantly different results when performed on combined bone and cartilage segmentations derived from MRI scans? and (3) Does the amount of cartilage in the joints have a significant correlation to the differences observed between planning on bone or combined bone and cartilage segmentations?

## Materials and methods

Automated osteotomy planning was performed in threefold for each patient, to study the effect of different scanning modalities and tissue inclusion on the outcome. The planning was performed once using bone segmentation derived from CT, once using bone segmentations derived from MRI, and once using bone and cartilage segmentations from MRI. As cartilage was not discernible on the CT scans, cartilage segmentation was not performed on CT scans.

### Data

The data were acquired prospectively at the Erasmus MC (Erasmus University Medical Center, Rotterdam, The Netherlands) under ethical approval of the Medical Ethical Testing Committee, reference number 52987.078.15. Detailed information on the inclusion criteria and scan parameters can be found in the study by Roth et al. [12] and in the Supplementary Material S.1.

Of the eighteen patients included in this study, eight patients had to be excluded due to irregularities such as movement during scanning, incomplete coverage of the region of interest or implant-induced artifacts. Additionally, the CT scan of Patient 6 was incomplete and thus only the MRI of this patient was used. The demographics of the patients are shown in Table 1.

**Table 1:** Demographics of the patients and age at time of trauma, scan, and the interval between the trauma and scan

Patient	Sex	Side trauma	Age (years)		
			Trauma	Scan	Interval
1	F	Right	7.1	20.1	13.0
2	M	Left	7.6	13.9	6.3
3	M	Left	9.2	11.9	2.7
4	F	Left	9.7	10.7	1.0
5	M	Left	17.6	18.3	0.7
6	M	Left	14.0	22.9	8.9
7	F	Left	10.7	20.1	9.4
8	M	Left	13.7	14.6	0.9
9	M	Left	7.4	12.3	4.9
10	M	Right	14.8	16.6	1.8
Mean	–	–	11.2	16.1	5.0
Std. Dev.	–	–	3.4	3.9	4.1

### Segmentation

**Fig. 1:**
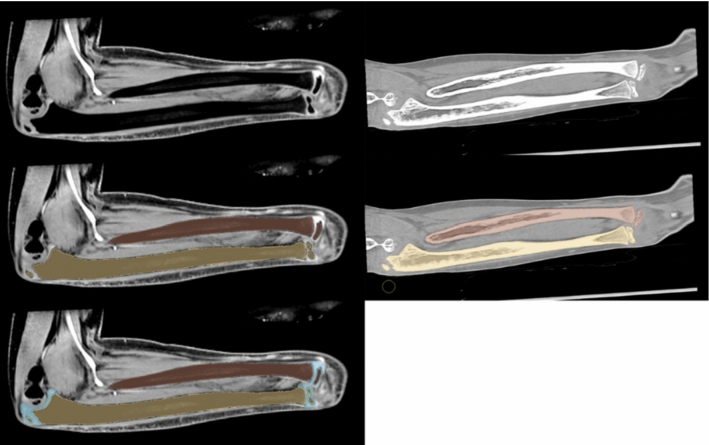
Top row: example of the MRI (left) and CT (right) of lower right arm. Middle row: MRI and CT overlaid with radius (brown) and ulna (yellow) bone segmentation. Bottom row: MRI overlaid with bone and cartilage (blue) segmentation

**Fig. 2:**
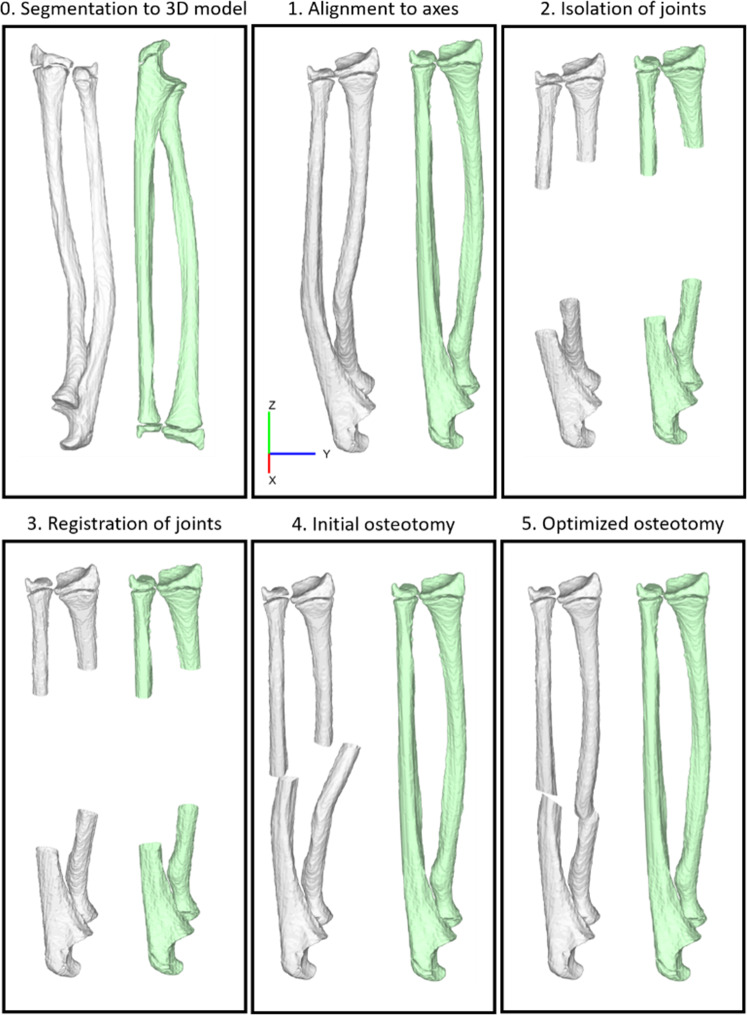
Step-by-step overview of the automatic osteotomy planning workflow. The deformed radius and ulna (white) were aligned to the mirrored healthy contralateral radius and ulna (green) by registering the joints and subsequently optimizing the osteotomy plane to minimize the distance between the proximal and distal bone fragments. This example used a bone segmentation derived from MRI

Before segmentation, both CT images and MRI images were resampled to isotropic 0.5 × 0.5×0.5 mm^3^ voxels using trilinear interpolation. The radius and ulna were manually segmented from the CT and MRI scans to produce two sets of 3D bone models for each patient. Additionally, articular cartilage was manually segmented from the MRI images to produce 3D cartilage models. One experienced biomedical engineer (RK) performed all segmentations. A detailed description of the segmentation process can be found in Supplementary Material S.1. In the remainder of the text, the segmentation of bone from the CT and MRI scans will be referred to as CTb and MRb, and the segmentation of both bone and cartilage from the MRI scan will be referred to as MRbc. Examples of the CTb, MRb, and MRbc segmentation are shown overlaid on the CT and MRI scans in Fig. 1. After segmentation, all bone models were transformed from a voxel-wise representation into vertex-and-edge-based triangulated surface mesh (Fig. 2—step 0).

### Automatic planning

The automatic planning was performed using a novel in-house produced software tool, which was inspired by existing methods such as described by Caiti et al., Dobbe et al., and Carrillo et al. [7, 10, 11]. It used the CTb, MRb, and MRbc segmentations to perform the osteotomy planning in five automatic steps, which are summarized in Fig. 2.

First (Fig. 2—step 1), the CTb, MRb, and MRbc models are all aligned. Second (Fig. 2—step 2), the proximal and distal part of the deformed and mirrored healthy contralateral bone were isolated. Third (Fig. 2—step 3), these deformed fragments were each registered separately to their counterparts on the healthy contralateral bone, resulting in four 4 × 4 homogenous transformation matrices for each bone, ${M}_{\mathrm{prox}}^{\mathrm{radius}}$ and ${M}_{\mathrm{dist}}^{\mathrm{radius}}$, for the distal and proximal radius, and ${M}_{\mathrm{prox}}^{\mathrm{ulna}}$ and ${M}_{\mathrm{dist}}^{\mathrm{ulna}}$, for the ulna. Fourth (Fig. 2—step 4), the original deformed bone model was osteotomized into two parts using the plane P1. This plane was defined by six parameters: a center point P1p = (P1x, P1y, P1z) located in the center of the bone in the axial plane, and rotation of the plane around the principal axes, P1r = (P1rx, P1ry, P1rz). The saved transformations ${M}_{\mathrm{prox}}^{\mathrm{radius}}$, ${M}_{\mathrm{dist}}^{\mathrm{radius}}$, ${M}_{\mathrm{prox}}^{\mathrm{ulna}}$, and ${M}_{\mathrm{dist}}^{\mathrm{ulna}}$ were used to reconstruct the proximal and distal fragment into the desired positions, aligned with the mirrored healthy bone. Finally, in the fifth step (Fig. 2—step 5), an exhaustive search method was employed to find the optimal location and orientation of the osteotomy.

The automatic planning was performed for each of the three segmentations separately: CTb, MRb, and MRbc. A more detailed description of each step of the automatic osteotomy planning process can be found in Supplementary Material S.2.

### Evaluation

The differences in segmentation between the CTb and MRb were calculated from the 3D bone models, after the initial alignment (Fig. 2—step 2, Supplementary Material S.2). They were quantified by four metrics, computed as described by Taha and Hanbury [13]: the Dice Similarity Coefficient (DSC), the Mean Absolute Surface Distance (MASD), the Hausdorff Distance (HD), and the 95th percentile Hausdorff Distance (HD95). The relative cartilage volume (RCV) was calculated separately for the radius and ulna, and was defined as the cartilage volume divided by the bone volume as segmented from MR.

Bone realignment was defined as the rotational and translational difference between the distal bone fragment relative to the proximal bone fragment. The difference was calculated by finding the difference between the two transformation matrices that defined the bone reconstruction, using:

$${M}^{\mathrm{radius}}{{=M}_{\mathrm{prox}}^{\mathrm{radius}}}^{-1}\times {M}_{\mathrm{dist}}^{\mathrm{radius}}\,\mathrm{and }\,{M}^{\mathrm{ulna}}{{=M}_{\mathrm{prox}}^{\mathrm{ulna}}}^{-1}\times {M}_{\mathrm{dist}}^{\mathrm{ulna}}$$

Differences in relative rotational and translational realignment due to the use of differing segmentations could then be calculated as follows:

$${M}_{CTb-MRb}^{\mathrm{radius}}{{=M}_{CTb}^{\mathrm{radius}}}^{-1}\times {M}_{MRb}^{\mathrm{radius}}\, \mathrm{and }\,{M}_{MRb-MRbc}^{\mathrm{radius}}{{=M}_{MRb}^{\mathrm{radius}}}^{-1}\times {M}_{MRbc}^{\mathrm{radius}}$$

$${M}_{CTb-MRb}^{\mathrm{ulna}}{{=M}_{CTb}^{\mathrm{ulna}}}^{-1}\times {M}_{MRb}^{\mathrm{ulna}}\, \mathrm{and }\,{M}_{MRb-MRbc}^{\mathrm{ulna}}{{=M}_{MRb}^{\mathrm{ulna}}}^{-1}\times {M}_{MRbc}^{\mathrm{ulna}}$$

A transformation matrix can be transcribed to translations (Δx_**S**_, Δy_**S**_, and Δz_S_) and Euler angle rotations (Φx_**S**_, Φy_**S**_, and Φz_**S**_) along the three orthogonal axes, with **S** either CTb, MRb, or MRbc. The total translation and rotation were then defined as T_**S**_ = √(Δx_S_^2^ + Δy_S_^2^ + Δz_S_^2^) and R_**S**_ = √(Φx_S_^2^ + Φy_S_^2^ + Φz_S_^2^). The Shapiro–Wilks test was used to check whether differences in T and R between the methods were normally distributed, and if so, two-tailed paired t-tests were performed to check for significant mean differences. Differences between two methods were also calculated as the Euclidean translational distance ΔT = √((Δx_S1_–Δx_S2_)^2^ + (Δy_S1_–Δy_S1_)^2^ + (Δz_S1_–Δz_S2_)^2^) and rotational distance Φ*R* = √((Φx_S1_–Φx_S2_)^2^ + (Φy_S1_–Φy_S2_)^2^ + (Φz_S1_–Φz_S2_)^2^), which is suitable for rotations where: Φx, Φz ∈ [− π,π) and Φy ∈ [− π/2,π/2) radians [14].

To estimate whether the differences in planning with the various segmentations were clinically relevant, we compared these with the residual error after surgery, as found by Vlachopoulos et al. [15]. As the definition of the axes used in this study did not directly correspond to ours, the total rotational (5.6º ± 4.2º) and translational (2.0 ± 1.4 mm) residual errors were used.

Finally, we compared the osteotomy planning by location and rotation of the cutting plane. The location was defined as the distance from the proximal end of the bone to the center of the osteotomy, along the axis of the bone, and denoted as ΔZ. The osteotomy orientation was defined by the rotation of the osteotomy plane around the two axes *ψ*X and *ψ*Z, perpendicular to the longitudinal axis of the bone.

## Results

### Segmentation accuracy

**Fig. 3:**
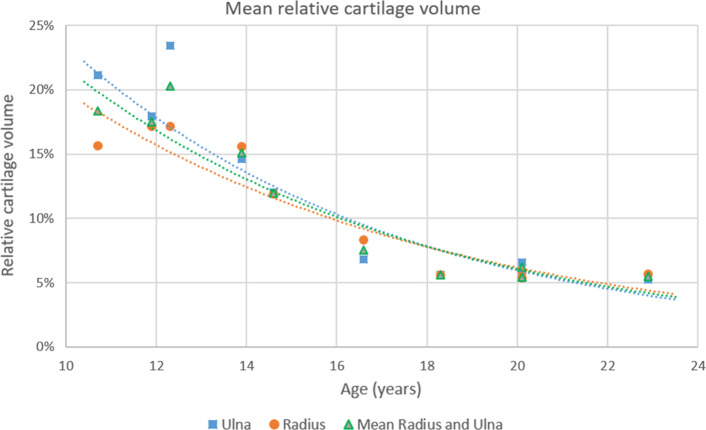
Relative cartilage volume against the age of each patient at the time of the MRI scan. Both cartilage and bone segmentation were acquired from the MRI scan. An exponential regression line is fitted to show the decrease in relative cartilage volume by age

An example of the CT and MRI segmentations is shown in Fig. 1. The difference between MRI- and CT-derived bone segmentation is summarized in Table 2. The RCV was plotted against the age of the patients in Fig. 3. Exponential regression lines have been fitted to the data to illustrate the diminishing RCV with age.

**Table 2:** Average segmentation differences between CT and MRI bone segmentation. DSC = Dice Similarity Coefficient, MASD = Mean Absolute Surface Distance, HD = Hausdorff Distance, and HD95 = 95th percentile Hausdorff Distance

	DSC	MASD (mm)	HD (mm)	HD95 (mm)
Mean	0.95	0.41	4.36	0.86
Std. Dev	0.02	0.07	2.38	0.28

	Ulna	Radius	Ulna	Radius	Ulna	Radius	Ulna	Radius
Mean	0.95	0.95	0.41	0.42	4.32	4.40	0.86	0.85
Std. Dev.	0.02	0.02	0.06	0.08	2.32	2.44	0.20	0.33

### Bone realignment

The bone realignment was performed in threefold, using the CTb, MRb, and MRbc segmentations. In Table 3, the mean difference in realignment when using the CTb, MRb, and MRbc segmentations is shown. For all methods, the differences between the total translational (T) and rotational (R) realignment parameters were found to be normally distributed. Significant mean differences were found between T_CTb_ and T_MRb_ (*p* = 0.026) but not between R_CTb_ and R_MRb_ (*p* = 0.831). Significant differences were also found between T_MRb_ and T_MRbc_ (*p* = 0.031) but not for R_MRb_ and R_MRbc_ (*p* = 0.173). The intraclass correlation coefficient (ICC) was calculated using the two-way random model for absolute agreement of single measures, denoted as ICC(3,1). The translation and rotation for all comparisons was larger than 0.949, corresponding to excellent reliability (ICC > 0.9) [16].

**Table 3:** Mean difference and ICC for the relative translation and rotation between the different methods of planning; on CTb, MRb, and MRbc. ICC(3,1): two-way random model for absolute agreement of single measures was used [16]

	CTb and MRb		MRb and MRbc	
	ΔT (mm)	ΦR (º)	ΔT (mm)	ΦR (º)
Mean	4.5	4.0	2.8	2.7
Std. Dev	2.1	2.4	2.1	2.6

	Δx	Δy	Δz	Φz	Φy	Φx	Δx	Δy	Δz	Φz	Φy	Φx
Mean	1.1	0.7	− 0.3	0.2	− 0.3	0.2	1.3	− 0.1	0.2	1.0	− 0.3	− 0.1
Std. Dev.	2.6	3.9	0.7	4.3	0.7	1.5	2.5	1.9	0.6	3.5	0.7	0.7
ICC	0.99	0.99	0.99	0.95	0.97	0.96	0.99	0.99	0.99	0.99	0.99	0.99

**Fig. 4:**
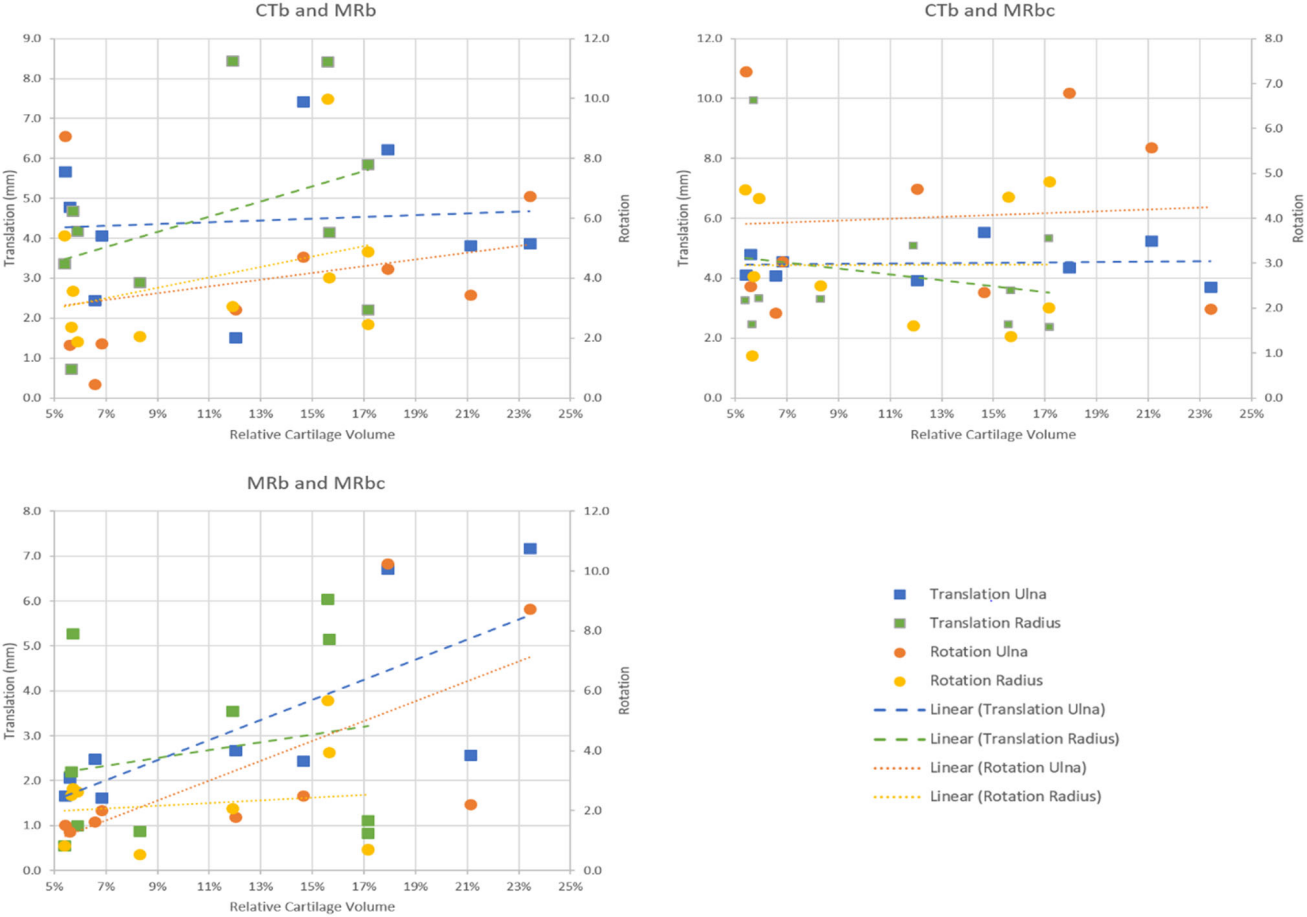
Translational and rotational realignment differences when planning the osteotomy using CTb, MRb, and MRbc, plotted against the relative cartilage volumes of the patients. Linear trendlines have been fitted

In Fig. 4, the differences in realignment when using different segmentations are shown, plotted against the RCV of the patient. Moderate-to-strong correlations were only found between the RCV and the difference in rotational (*R* = 0.68, *p* = 0.043) and translational (*R* = 0.74, *p* = 0.024) realignment of the ulna when using MRb versus MRbc. Only weak correlations (|*R*|< 0.3) were found between the RCV and the realignment differences between CTb versus MRb and CTb and MRbc.

**Fig. 5:**
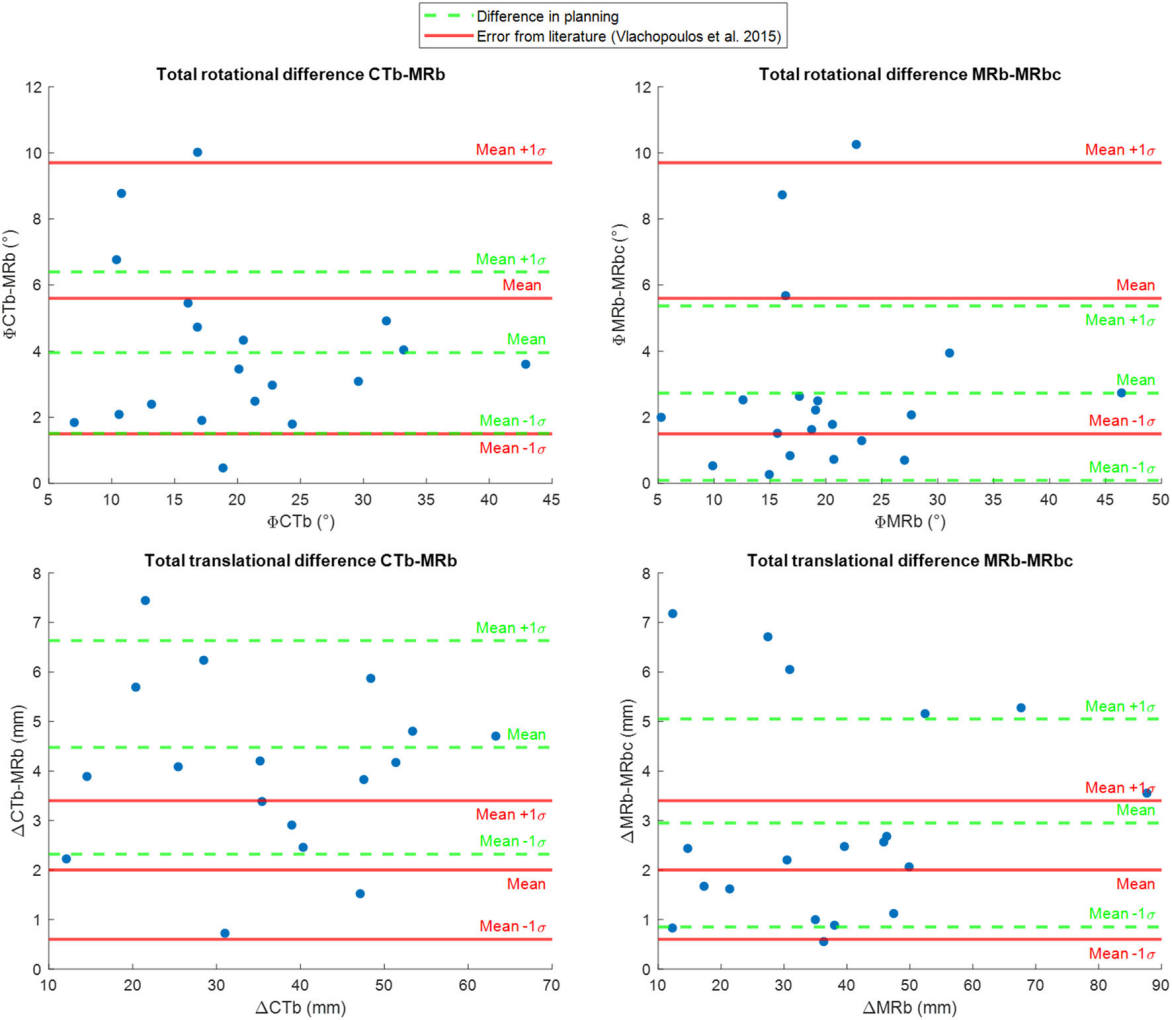
The rotational (top) and translational (bottom) differences between the CTb and MRb (left) and MRb and MRbc (right) in relative bone realignment after osteotomy are shown. The mean and standard deviation of the differences is compared to residual error after osteotomy surgery as reported by Vlachopoulos et al. [24].

**Fig. 6:**
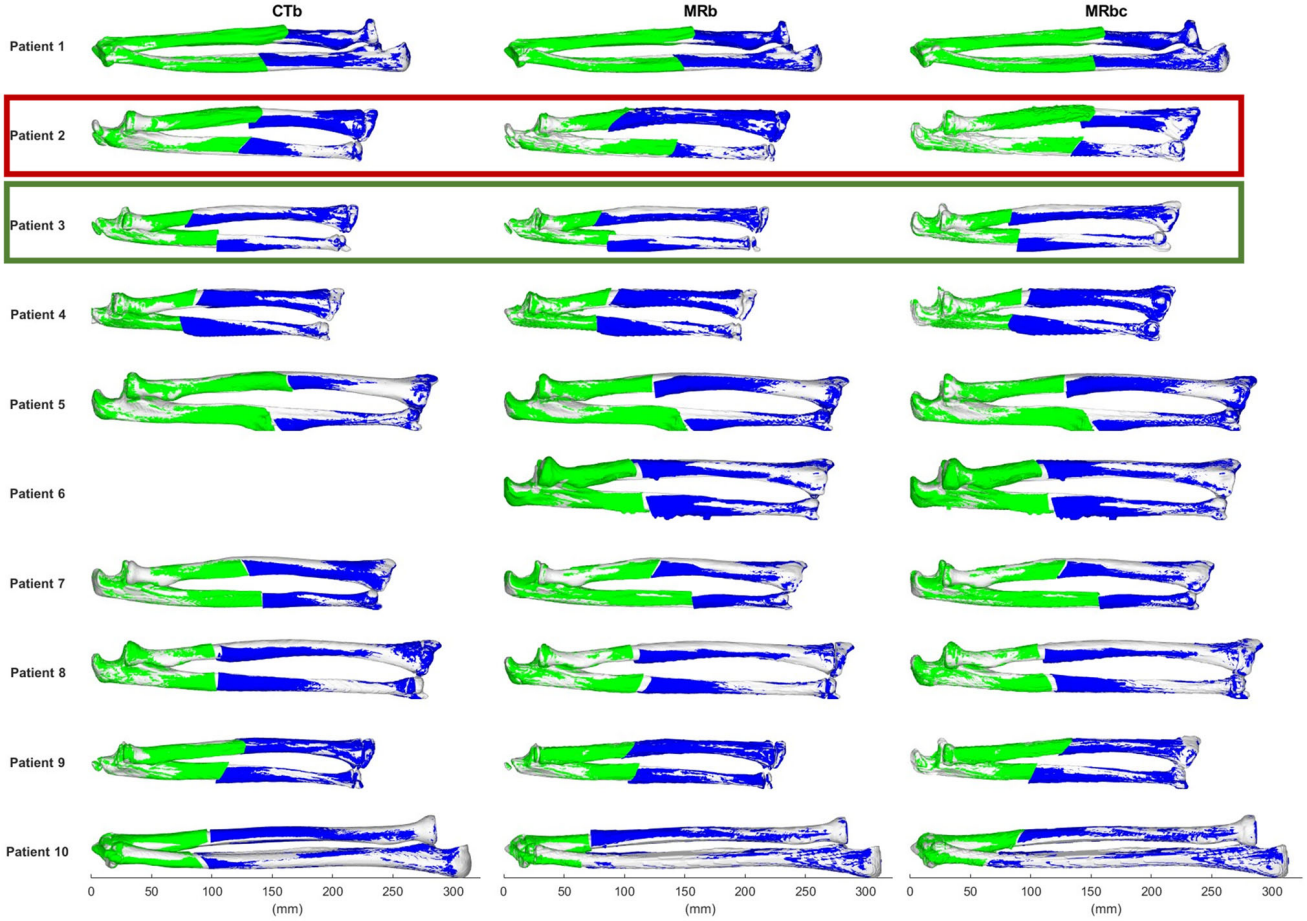
Illustration of the difference in simulated bone reconstruction in the radius and ulna between CTb, MRb, and MRbc segmentations for all patients. The proximal (blue) and distal (green) fragment of the deformed bone after the planned reconstruction are overlayed on the mirrored healthy contralateral side (white). The CT scan of Patient 6 was incomplete and thus not included. Red rectangle: patient with largest relative rotational difference in ulna between MRb and MRbc. Green rectangle: patient with largest relative translational difference in ulna between MRb and MRbc

The graphs in Fig. 5 show that the rotational differences in realignment were on average smaller than the residual errors that were found after osteotomy surgery in a clinical study by Vlachopoulos et al. [15], but the translational errors were larger. Visual comparisons of the final bone reconstructions are shown in Fig. 6.

### Osteotomy plane optimization

**Fig. 7:**
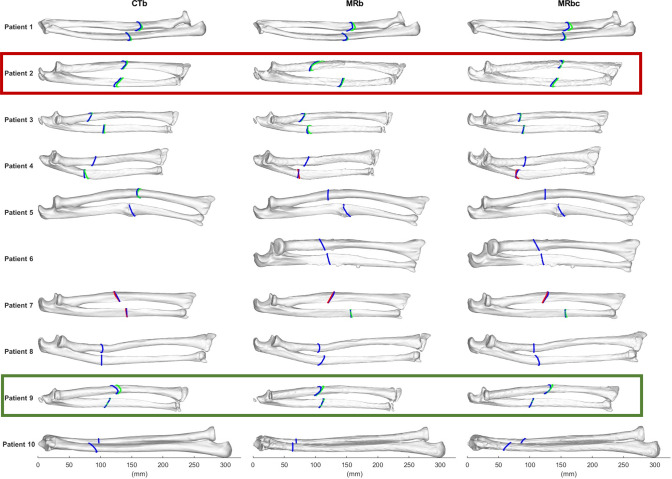
Illustration of the difference in optimal computed osteotomy location and orientation in the radius and ulna between CTb, MRb, and MRbc segmentations for all patients. The blue line indicates the primary osteotomy cut. Where necessary, the secondary osteotomy cut is visible. The secondary cut is shown in green if it does not intersect the primary cut, or in red if it does overlap the primary cut. The CT scan of Patient 6 was incomplete and thus not included. Red box: patient with largest difference in (radius) osteotomy location between MRb and Mbc. Green box: patient with highest RCV

**Fig. 8:**
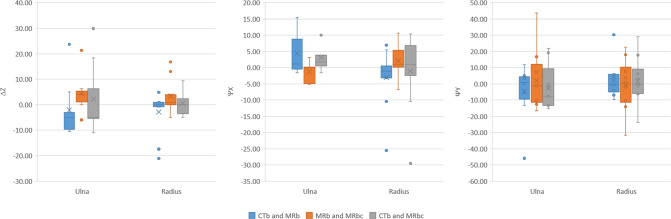
Differences in location (ΔZ) and orientation (*ψ*X and *ψ*Z) of the osteotomy plane between the different methods of planning; on CTb, MRb, and MRbc

A visual comparison of the osteotomy plane location and orientation on the CTb, MRb, and MRbc is shown in Fig. 7. The mean differences that arise due to the planning on different segmentations are summarized in Table 4, and the distribution of these metrics for both the ulna and radius is shown in Fig. 8.

**Table 4:** Mean difference in rotation and location of the osteotomy plane between the different methods of planning; on CTb, MRb, and MRbc

	CTb and MRb			CTb and MRbc			MRb and MRbc		
	ΔZ (mm)	*ψ*Y(º)	*ψ*Z (º)	ΔZ (mm)	*ψ*Y(º)	*ψ*Z (º)	ΔZ (mm)	*ψ*Y(º)	*ψ*Z (º)
Mean	− 2.5	0.5	− 1.0	3.8	0.4	0.6	1.3	0.9	− 0.5
Std. Dev.	9.3	8.6	13.9	6.6	4.4	16.4	9.5	8.8	13.6

## Discussion

The aim of this study was to evaluate differences in radio–ulnar osteotomy planning based on CT- and MRI-derived segmentations of the bone and/or cartilage. Although the realignment parameters showed excellent correlation, statistically and clinically significant mean differences were found, both due to segmentation differences of the bone between CT and MRI scans, as well as due to differences between planning on bone and bone with cartilage.

A prerequisite for this study was an accurate method for the segmentation of bone and cartilage. The segmentation of CT scans was relatively straightforward, as bone is generally the only tissue with intensity values over 200 HU. However, issues such as partial volume effects, small joint spaces, and artifacts due to foreign objects necessitated at least some manual correction in all CT segmentations.

Segmentation of bone from MRI scans was more complicated, as there was no unique intensity range for bone that separated it from the other tissues. Additionally, the resolution of the MRI scans was lower than the CT scans. Still, the results showed that the MASD between MRI and CT bone segmentation was approximately equal to the resampled voxel size of the segmentations, at 0.41 mm. For comparison, the most relevant reference in the literature for comparable segmentations was Marin et al. [9], who reported an average MASD of 1 mm between forearm bones that were manually segmented from CT and MRI.

Segmentation of the cartilage from MRI scans was entirely manual, which caused it to be a time-consuming process. This increases the risk of low intra- and interobserver agreement between segmentations, as noted by Brui et al. [17], who observed this when segmenting wrist cartilage from MRI (DSC = 0.9 intra-observer and DSC = 0.78–0.88 interobserver). For an efficient, deterministic clinical workflow, the issues with manual segmentation from MRI would need to be addressed, for example, by employing cartilage-specific MRI acquisition sequences as proposed by Dalili et al. [18], Heckelman et al.[19], and Zink et al.[20].

The first hypothesis of this study was that automated osteotomy planning would yield comparable results when performed using CT and MRI. Excellent correlation (ICC(3,1) > 0.9) was found for each of the translational and rotational realignment components. However, the differences in realignment showed a significant mean difference of the total translational component T, but no significant mean difference in the total rotational component R. Larger differences were also seen for the osteotomy plane localization (Δ*Z* = 6.7 ± 6.9 mm) and orientation (*ψY* = 5.5 ± 6.7º and *ψZ* = 8.4 ± 11.1º). This indicated that the impact of using the CTb and MRb had a relatively small effect on the rotational realignment of the bone, but had a greater impact on the translational realignment and the position and orientation of the osteotomy plane.

The second hypothesis of this study was that the same method of osteotomy planning would yield significantly different results when cartilage was included. Again, the translational and rotational components of the realignment showed excellent correlation. Significant mean differences were found for the translational, but not for the rotational realignment. The results in Table 4 and Fig. 8 showed that the differences between MRb and MRbc in osteotomy plane localization (Δ*Z* = 6.5 ± 7.0 mm) and orientation (*ψY* = 5.9 ± 6.6º and *ψZ* = 10.8 ± 8.4º) were similar to the differences between CTb and MRb. The mean difference in realignment between planning on CTb and MRb was slightly larger than the difference in planning between MRb and MRbc. This means that the impact of segmentation differences between modalities was on average larger than the impact of including cartilage.

Comparison with the residual errors found after surgery showed that the differences in planned translational realignment were larger than the accuracy with which osteotomy surgery is performed. During surgery, the pre-operative planning is assumed to be the optimal reconstruction. Variations in planning due to the use of different planning methods would induce larger uncertainty in surgery outcome and should, therefore, ideally have a significantly smaller variation than the surgery itself. The large differences between CTb, MRb, and MRbc planning observed here would, therefore, have an undesirable, clinically significant impact on the outcome, and should, thus, not be ignored.

The third hypothesis stated that the relative amount of articular cartilage would be correlated with the effect cartilage inclusion has on the osteotomy planning. Firstly, we found that the RCV was higher in younger adolescents and decreased with age, which corresponds to the established process of endochondral ossification [21]. A positive correlation was also found between the RCV and ulnar translational differences between MRb and MRbc. This indicated a larger effect of cartilage inclusion in younger patients, who generally have a higher RCV, than in older patients. This effect was only present in the ulna, possibly due to the differences in the endochondral ossification process of each bone [21]. It is important to note that this does not show that inclusion of cartilage information would improve the outcome of surgical intervention. It only shows that there is significant difference, of which the effect of clinical outcome is uncertain and should be further studied in clinical trials.

Using these three hypotheses, we found that small differences in segmentation and the inclusion of endochondral cartilage in the segmentation could have a significant impact when planning osteotomies for young patients. Roth et al. [22] found that performing osteotomy at a young age (< 13 years) was one of the prime predictors for better functional outcome. However, planning using CT carries an increased risk of radiation-induced diseases, especially in young patients [23]. Further research into the applicability and impact of using MRI for the planning of osteotomies could, therefore, be of great interest.

## Limitations

A limitation of this study was that the tool might not have incorporated all the criteria that an orthopedic surgeon considers during the planning of an osteotomy. It found the optimal solution to the objective function that it was presented with, which included only two optimization targets: increase the amount of volume overlap with the healthy contralateral example and minimize the distance between the osteotomy surfaces. Other constraints that might be considered include the proximity of important tissues such as tendons, muscle, and blood vessels, the presence of earlier implants or incision scars, and the relation between the osteotomy locations on the radius and ulna.

It should also be noted that the reconstruction was constrained to optimal realignment of the deformed joints to the mirrored contralateral side. Therefore, only a closing and opening wedge osteotomy were considered. Other techniques, such as the oblique single- or double-cut rotational osteotomy [24], might have resulted in a different planning.

Due to the difficulty of obtaining a large dataset that adheres to the narrow inclusion criteria that were used, and to obtain both CT and MRI scans of both the deformed and healthy forearms, the dataset was relatively small and scanner settings were inhomogeneous. For a perfect comparison, the voxel sizes of all CT and MR scans should have been the same to avoid differences in planning due to differences in scanner precision. Additionally, a study based on a larger dataset would have more power and could, thus, more accurately prove or disprove the hypotheses. Finally, intra- and interobserver segmentation differences of bone and cartilage from CT and MRI were not included in this study. As discussed previously, especially MRI segmentation could be subject to significant intra- and interobserver segmentation differences. The impact of this would be an important subject of further study.

## Conclusion

In this study, we performed automatic radius and ulna osteotomy planning based on bone and/or cartilage segmentations from CT and MRI. Excellent correlations were found between the realignment parameters when comparing CT and MRI segmentations, and when comparing MRI bone and bone and cartilage segmentations. However, statistically significant mean differences were found in the translational component of the bone realignment for both methods, which were larger than the differences reported in the literature between planned and realized osteotomy surgery. This indicated that small differences in segmentation might have a clinically significant impact on the osteotomy planning.

When we compared planning on bone and bone with cartilage, we found a positive correlation between the relative cartilage volume of the patient and the difference in realignment of the ulna. This indicated that endochondral cartilage might also be a non-negligible factor when planning osteotomies for young patients.

### Supplementary Information

Below is the link to the electronic supplementary material.

Supplementary file1 (DOCX 27 KB)

## Data Availability

Data acquisition for this study was performed Erasmus University Medical Center (Rotterdam, The Netherlands) under ethical approval of the Medical Ethical Testing Committee, reference number 52987.078.15. Data processing, evaluation, and writing of the manuscript were performed at the UMCU (Utrecht, The Netherlands). It is available on the WHO Platform under reference NL8059 and can be found at: https://trialsearch.who.int/Trial2.aspx?TrialID=NL8059
