# Supplementary material for: Impact of bone and cartilage segmentation from CT and MRI on both bone forearm osteotomy planning
Source: Int J Comput Assist Radiol Surg. 2023 May 23;18(12):2307–18. doi: 10.1007/s11548-023-02929-8 (PMC10632286; doi:10.1007/s11548-023-02929-8)
Supplement: Supplementary file 1 — Supplementary file1 (DOCX 27 KB) [file 11548_2023_2929_MOESM1_ESM.docx]

# Supplementary Material

## S.1 Inclusion criteria and acquisition parameters

The inclusion criteria and acquisition parameters were described earlier by Roth et al [1] and were as follows:

Inclusion criteria:

- A diaphyseal both-bone forearm fracture sustained during childhood (younger than 18 years).
- Minimum age of 10 years at time of injury.
- Pronation or supination range of motion of less than 50°.
- Unsatisfactory improvement after conservative treatment.

Exclusion criteria were:

- A traumatic osseous deformity of the contralateral forearm.
- Congenital or developmental deformity of the contralateral or affected forearm.

For each patient, CT and MRI scans were taken on the same day, of both forearms. The CT scans of both arms were taken using a single scan, with the patient in prone position and the shoulders and elbows in maximal extension (Superman position). The CT scans were acquired on a dual-source scanner plane (SOMATOM Force, Siemens Healthcare AG, Erlangen, Germany) with 0.6 mm axial slice thickness and pixel spacing ranging from 0.39 to 0.87 mm. The tube current was 89 mA with 120 kV tube voltage.

MRI scans were taken separately for each arm, with the arm positioned above the head and elbow in 90-degree flexion. MRI scans were acquired in a 3T scanner using a T1-weighted Multi-echo Gradient-echo sequence (GE Healthcare, Chicago, IL, USA) using a 3D imaging mode. Dixon reconstruction was performed to obtain in-phase (IP), opposed phase (OP), fat only (F), and water only (W) images [2]. The acquisition time was 3 min 21 s with TR = 6.64 ms, TE = 2.97 ms, bandwidth = 325.5 Hz/pixel, flip angle = 15°, average acquired voxelsize = 0.66 × 0.66 × 1 mm^3^. A 35% phase field of view was used to reduce scan time. The Field of View of the scan was on average 512x512x324 voxels, or 337x337x324 mm.

## S.1 Segmentation

Each bone was segmented separately from the CT scans using the open source medical imaging computing platform 3D Slicer [3]. First, a threshold of 200 Hounsfield Units (HU) was applied and the largest component corresponding to the bone of interest was manually selected. Additional manual editing was performed to fill holes in the outer shell of the bone or to remove spurious voxel segmentations caused by noise, hyper-intensities due to foreign materials, or connections between bones due to partial volume effects caused by low imaging resolutions. The medullary cavity of the bone was also filled such that a solid bone segmentation was acquired.

For segmentation of the MRI scans, only the water weighted Dixon reconstruction was used on which bone is visible as a low intensity region. Bias field correction was first performed using the N4ITK filter[4], available in 3D Slicer. The parameters used for the N4ITK filter were: mask image = none, BSpline grid resolution = 5x5x5, number of iterations = 50, 40, 30, convergence threshold = 0.0001, BSpline order = 3, shrink factor = 4. The separate bones on MRI scans were then coarsely segmented by using a local inverse Otsu-threshold [5] on a manually delineated area that included bone and soft tissue, but no background. The bone segmentation was refined by manually removing parts of the segmentation that were not part of the bone. The cartilage on the joint ends of each bone was then segmented entirely by manual delineation of the cartilage outline by an experienced operator, as there was not one clear intensity threshold that separated it from the cartilage of the adjacent bones and surrounding tissue.

## S.2 Automatic Planning

The automatic planning was performed using an in-house produced software tool written in Matlab (MATLAB 2020a, The MathWorks, Inc., Natick, Massachusetts, United States). It used the CTb, MRb and MRbc segmentations to perform the osteotomy planning in five automatic steps, which are summarized in Figure 2. Figure 2 – step 0 shows the original MRb segmentation of one of the patients before the osteotomy planning, before the deformed and healthy bone models were aligned.

First (Figure 2 – step 1), we aligned the CTb, MRb and MRbc models. To align the arms of all patients along approximately the same directions, the principal components of the combined point-clouds of the healthy CTb and MRb radius and ulna bone models were calculated. The healthy CTb and MRb bone models were then rotated such that the Z, X and Y axes of the patient were defined as the first, second, and third principal components respectively. The MRbc was rotated using the same transformation as the MRb, such that their relative orientation remained the same. Next, for each patient, the 3D models from the CTb radial and ulnar bones were separately registered to the same bones from the MRb using Iterative Closest Point [6] (ICP) matching. This ensured that the CTb, MRb and MRbc were all aligned similarly.

Second (Figure 2 – step 2), the proximal and distal part of the deformed and mirrored healthy contralateral bone were isolated. The proximal and distal joint were separated from the rest of the bone at a distance equal to 30% of the total length of each bone, measured from their respective joints. This prevented the site of the deformity to be taken into account in the reconstruction, while providing the registration algorithm with enough bone surface to converge to a realistic solution.

Third (Figure 2 – step 3), these deformed proximal and distal bone fragments of the radius and ulna were each registered separately to their counterparts on the healthy contralateral bone using ICP. This produced four 4x4 homogenous transformation matrices for each bone, $M_{prox}^{radius}$ and $M_{dist}^{radius}$, for the distal and proximal radius, and $M_{prox}^{ulna}$ and $M_{dist}^{ulna}$, for the ulna. For each reconstruction, optimal realignment of the proximal and distal joints to the mirrored contralateral side was set as a constraint. Due to this, an opening wedge osteotomy was necessary in case of bone lengthening, and a closing wedge osteotomy in case of bone shortening.

Fourth (Figure 2 – step 4), the original deformed bone model was osteotomized into two parts at a certain location along the bone, perpendicular to the long axis of the bone, using the plane P1. This plane was defined by six parameters: a center point P1p = (P1x, P1y, P1z) located in the center of the bone in the axial plane, and rotation of the plane around the principal axes, P1r = (P1rx, P1ry, P1rz). The saved transformations $M_{prox}^{radius}$, $M_{dist}^{radius}$, $M_{prox}^{ulna}$ and $M_{dist}^{ulna}$ were used to reconstruct the proximal and distal fragment into the desired positions, aligned with the mirrored healthy bone. In case bone overlap between the proximal and distal part occurred after the digital reconstruction, the overlapping part of the bone had to be removed by a second osteotomy plane P2. The location and normal vector of this plane were found by first transforming the plane P1 forward using the transformation $M_{prox}^{radius}$ or $M_{prox}^{ulna}$, and then backward using the inverse of $M_{prox}^{radius}$or $M_{prox}^{ulna}$. In case bone was present between P1 and P2 there was an overlap of bone after reconstruction. Only then this second osteotomy plane was necessary, and the bone between P1 and P2 would be removed.

Finally, in the fifth step (Figure 2 – step 5), an exhaustive search method was employed to find the optimal location and orientation of the osteotomy that minimized a predefined objective function. Although the location and orientation of the plane were defined by six parameters, only three parameters were used to search the solution space. The location of the center point of the plane was only dependent on the location along the longitudinal direction (P1z) of the bone, as P1x and P1y were constraint to be along the centroid of the bone in the x-y plane. The normal vector was dependent only on the rotation along the directions perpendicular to the longitudinal axis of the bone (P1rx and P1ry), as possible orientations could be defined using those two rotations. The search space for the location (P1z) was constraint to the diaphysis of the bone, between 20% and 80% of the length, and between angles of -35° and +35° in both directions for the orientation (P1rx and P1ry).

The objective function was defined as the weighted (w_1_, w_2_) average of two metrics. The first metric (m_1_) quantified the distance between the osteotomy planes after reconstruction, defined as the mean squared minimal distance between all points on the edge of the proximal osteotomy surface to the distal osteotomy surface. The second metric (m_2_) quantified the degree to which bone protrudes outwards after reconstruction, defined as the minimal squared distance between all points on the reconstructed bone to the healthy contralateral template. The metrics were equally weighted and thus w_1_ = w_2_ = 0.5. The objective function can then be written as:

$$\underset{P1z,P1rx, P1ry}{\arg\min} {(w}_{1}m_{1}+ w_{1}m_{2})$$

Subject to:

$$P1z\in\left[ 0.2,0.8 \right]$$

$$P1rx\in\left[ -35,35 \right]$$

$$P1ry\in\left[ -35,35 \right]$$

Step size for the exhaustive search was 1 mm for P1z and 1 degree for P1rx and P1ry. For each iteration of the exhaustive search step four of the automatic planning was repeated with new values for P1z, P1rx and P1ry and subsequently the objective function calculated.

This automatic planning was repeated three times, for each of the different bone models sets, i.e. bone segmented from CT, bone segmented from MR, and bone with cartilage segmented from MR. The resulting transforms and reconstructed bone models were saved to evaluate differences in osteotomy planning.

**References**

1. Roth KC, van Es EM, Kraan GA, Verhaar JAN, Stockmans F, Colaris JW (2021) Outcomes of 3-D corrective osteotomies for paediatric malunited both-bone forearm fractures. J Hand Surg Eur Vol 47:164–171. https://doi.org/10.1177/17531934211029511
2. Eggers H, Brendel B, Duijndam A, Herigault G (2011) Dual-echo Dixon imaging with flexible choice of echo times. Magn Reson Med 65:96–107. https://doi.org/10.1002/mrm.22578
3. Fedorov A, Beichel R, Kalphaty-Cramer J, Finet J, Fillion-Robbin J-C, Pujol S, Bauer C, Jennings D, Fennessy F, Sonka M, Buatti J, Aylward S, Miller J V, Pieper S, Kikinis R (2012) 3D Slicer as an Image Computing Platform for the Quantitative Imaging Network. Magn Reson Imaging 30:1323–1341. https://doi.org/10.1016/j.mri.2012.05.001.3D
4. Tustison NJ, Avants BB, Cook PA, Zheng Y, Egan A, Yushkevich PA, Gee JC (2010) N4ITK: Improved N3 bias correction. IEEE Trans Med Imaging 29:1310–1320. https://doi.org/10.1109/TMI.2010.2046908
5. Otsu N (1979) A Threshold Selection Method from Gray-Level Histograms. IEEE Trans Syst Man Cybern 9:62–66
6. Besl PJ, McKay ND (1992) A Method for the registration of 3-D Shapes. IEEE Trans Pattern Anal Mach Intell 14:239–256

**Supplementary Material for the manuscript titled:**
Impact of bone and cartilage segmentation from CT and MRI on both bone forearm osteotomy planning

**For the journal:**International Journal of Computer Assisted Radiology and Surgery

**Authors:**Ruurd J. A. Kuiper^1,2^, MSc
Joost W. Colaris^3^, MD, PhD
Filip Stockmans^4^, MD, PhD
Eline M. van Es^3^, MSc
Max A. Viergever^2^, PhD
Peter R. Seevinck^2^, PhD
Harrie Weinans^1^, PhD
Ralph J.B. Sakkers^1^, MD, PhD

1. Department of Orthopedics, University Medical Center Utrecht, Utrecht, The Netherlands
2. Image Sciences Institute, University Medical Center Utrecht, Utrecht, The Netherlands
3. Department of Orthopedics and Sports Medicine, Erasmus Medical Centre, Rotterdam, the Netherlands
4. Muscles & Movement, Department of Development and Regeneration, KU Leuven Campus Kulak, Kortrijk, Belgium

**Correspondence main author:**Department of Orthopedics
University Medical Center Utrecht
Heidelberglaan 100
3584 CX Utrecht
The Netherlands
Ruurdkuiper@gmail.com
